# Adhere to the Chinese dietary guidelines associated with better subjective well-being: evidence from a cross-sectional survey and a daily diary investigation

**DOI:** 10.1186/s12889-024-17880-9

**Published:** 2024-02-12

**Authors:** Jiaci Lin, Fuhua Yang, Miaosen Lan, Yichen Ding, Keli Yin

**Affiliations:** 1 Faculty of Education, Yunnan Normal University, Lianda Street, 650500 Kunming, Yunnan China; 2 School of Information, Yunnan University of Traditional Chinese Medicine, 650500 Kunming, China

**Keywords:** Chinese Dietary guidelines, Subjective well-being, Dietary patterns, Healthy diet

## Abstract

**Background:**

Subjective well-being (SWB) plays an essential role in general health. Although beneficial effects of selected micronutrients and foods on SWB have been reported, they do not reflect the impact of the habitual diet on SWB. Therefore, the purpose of this study is to investigate the association between adherence to the Chinese Dietary Guidelines (CDG) with SWB.

**Methods:**

This study combined a cross-sectional survey and a daily diary investigation. The cross-sectional survey was conducted on 1,433 students from 8 universities in southern China between October and November 2020. The daily diary investigation was conducted in November 2022, collecting 10-day daily data from 115 students at two universities in southern China over ten consecutive days, resulting in 1,020 valid matched daily responses. Adherence to the CDG was assessed by the China Prime Diet Quality Score (CPDQS), SWB was measured by the Index of Well-being (IWB) and the Affect Valuation Index (AVI). Correlation analysis, multiple regression analyses, and hierarchical regression were conducted to examine the associations of adherence to the CDG with its components, and SWB.

**Results:**

The cross-sectional survey revealed significant predictors of SWB, including adherence to the CDG (*β* = 0.24, *p*<0.001) and its components: cereals and tubers (*β* = 0.07, *p* = 0.024), vegetables and fruits (*β* = 0.11, *p*<0.001), dairy/soy/nuts (*β* = 0.11, *p* = 0.002), and condiments and alcoholic beverages (*β* = 0.08, *p* = 0.002). The daily diary investigation showed positive associations between adherence to the CDG (*β* = 0.19, *p*<0.001), and its components: vegetables and fruits (*β* = 0.11, *p* = 0.001), dairy/soy/nuts (*β* = 0.06, *p* = 0.009), animal source food (*β* = 0.06, *p* = 0.026), and condiments and alcoholic beverages (*β* = 0.07, *p* = 0.026), with higher levels of daily SWB.

**Conclusions:**

Adherence to healthy dietary patterns such as the CDG, rather than focusing on individual components in isolation, is associated with better SWB. Furthermore, the consumption of CDG components had an impact on SWB, although the specific effects varied between the two studies. This study offers modest evidence supporting the role of the CDG in promoting positive mental health.

## Background

Received wisdom as well as a large literature indicate that lifestyle has a substantial and highly significant impact on the health of individuals [1–3]. Among them, diet is a modifiable lifestyle behavior that contributes significantly to human health, accounting for 14% of its influence [4]. It surpasses medical factors (7%) and ranks second only to genetic factors (16%) [4]. Healthy dietary habits play a significant role in promoting mental and physical health [5], as well as in the prevention and treatment of non-communicable diseases [6]. Conversely, an unreasonable diet structure may lead to increasingly unhealthy conditions, including obesity [7], high blood pressure [8], high cholesterol [9], diabetes [10], and cancer [11].

In addition to physical functioning, health and quality of life benefits, diet is among the most important modifiable risk factors for mental health [12, 13]. An increasing number of studies have shown that various nutrients, food items, diet quality, and dietary patterns affect mental health [14, 15]. A systematic review has demonstrated that consuming fresh vegetables and fruits have been associated with a lower risk of depression, while sugar-sweetened beverages has been associated with a higher risk of depression [16]. Moreover, some research evidence points to the association between adherence to the Mediterranean dietary pattern and positive mental health outcomes including reduced anxiety, stress, and depression [17]. However, aside from exploring the relationship between diet and mental disturbances, the influences of a healthy diet on positive psychological functioning have not been extensively examined.

Subjective well-being (SWB) has consistently garnered scholarly attention and is frequently a sought-after state for individuals [18, 19]. SWB is a multifaceted construct, distinguishable into two primary components: affective and cognitive well-being [20–22]. Affective well-being pertains to the manifestation of positive affect (e.g., happiness) and the absence of negative affect (e.g., depressive mood). Cognitive well-being involves the cognitive assessment of overall life satisfaction and specific life domains. SWB is not only widely recognized as a crucial measure of a population’s overall health [23], but also plays a vital role in fostering satisfying interpersonal relationships, enhancing quality of life, and contributing to social productivity [21, 24]. While there is an essential role of SWB in overall health, there have been limited studies investigating its relationship with diet. Furthermore, unlike mental disorders such as depression and anxiety, well-being is less subject to heritable influences [25]. Therefore, modifying dietary factors is more likely to facilitate the enhancement of individual well-being [26]. A growing literature indicates that the consumption of healthy foods is associated with elevated levels of SWB [27–29]. An intervention study suggests that an 8-week period of providing participants with the recommended types and amounts of vegetables in the Dietary Guidelines for Americans led to an increase in SWB [30]. Additionally, the findings of a 30-day online survey indicated that increasing the daily intake of fruits and vegetables was associated with improved positive emotions, ultimately leading to a better mood [31]. On the other hand, the consumption of unhealthy foods, such as fast food and soft drinks, can indirectly undermine happiness [32].

Beyond single foods or nutrients [30, 33], examining overall dietary patterns is more likely to offer a comprehensive explanation of diet-SWB relations. It is widely recognized that individuals do not consume isolated nutrients but rather consume meals consisting of a diverse range of foods that contain complex combinations of interactive nutrients [34, 35]. Whole-diet analyses reflect real-life scenarios [26], depict a comprehensive picture of the interplay between foods and nutrients [36], and enable researchers to consider interactions among various nutrients [37, 38]. Thus, dietary patterns may be more predictive of SWB than foods or nutrients in isolation. Preliminary evidence indicates that healthy dietary patterns such as the Mediterranean diet, have been associated with enhanced subjective well-being [39, 40]. The Mediterranean diet is characterized by a substantial consumption of vegetables, legumes, fruits, nuts, cereals, and olive oil, with a moderate intake of wine, meat, fish, eggs, and dairy products, and a low intake of red meat, processed foods, and sugary drinks [41, 42].

To our knowledge, there has been limited exploration of the relationship between healthy dietary patterns and SWB, with the majority of the studies in the literature solely focused on the Mediterranean diet and limited by cross-sectional study design [39, 43]. Given the regional differences in diets around the world [44], conventional healthy dietary patterns such as the Mediterranean diet or Healthy Eating Index may not adequately align with the dietary structure of Chinese residents. The Chinese Dietary Guidelines (CDG) serves as official dietary guidelines specifically developed to promote healthy and habitual food choices, reduce the risk of chronic diseases, and enhance public health [45]. The CDG recommends a diet that emphasizes whole grains, vegetables, and fruits while advocating for moderate consumption of meat, poultry, eggs, and dairy products [45]. Previous research has extensively assessed the effects of adhering to the Chinese Dietary Guidelines (CDG) on physical and mental health [34, 46, 47]. However, the relationship between CDG adherence and SWB remains unexplored.

Therefore, the aim of the present study is to evaluate the association between adherence to the CDG and its components with evaluative SWB using a cross-sectional survey and a daily diary investigation. Considering the aforementioned health benefits of the healthy dietary pattern, we hypothesize that CDG adherence is also positively associated with SWB in the general population. The present study was approved by the Ethics Committee of ethics committee at Applied Psychology Program, Faculty of Education, Yunnan Normal University (2022010). Written informed consent form was obtained from all of the participants. The procedures conducted in this study adhered to the ethical standards set by the responsible committee on human experimentation (institutional and regional) and conformed to the principles outlined in the Helsinki Declaration of 1964 and all subsequent revisions.

## Materials and methods

### Study 1—the cross-sectional survey

#### Participants and procedure

From October to November 2022, a study was conducted among 1,452 Chinese college students from 8 public universities located in southern China. The study employed a convenience sampling method for data collection, utilizing a combination of online and on-site surveys. Investigators received standardized and uniform training, attaining proficiency in guiding language, questionnaire content, and precautions. Surveys were then conducted both online and on-site by assigned personnel at each school. Participants completed the questionnaires using either online links or hard-copy materials.

After the informed consent process, participants answered some basic demographic questions and self-reported weight and height information. Subsequently, self-reported data on adherence to the CDG and SWB were collected. Questionnaires were counterbalanced in terms of their order to mitigate potential order biases. Following exclusion of individuals without demographic information the remaining 1433 participants aged 17 to 28 years old (average age: 19.19 ± 1.32, 57.0% female) were included for analysis.

### Study variables and measures

Adherence to the Chinese Dietary Guidelines was assessed with the China Prime Diet Quality Score (CPDQS) [48]. The CPDQS is a food-based score designed to reflect the diet quality of individuals based on adherence to the 2016 Chinese Dietary Guidelines [48]. Dietary intakes of individuals, based on an average of 24-h recalls and brief questionnaire items, were scored according to 22 food groups. These groups encompass 15 recommended dietary components (dark green leafy vegetables, deep orange, other vegetables vegetables, deep orange fruits, citrus fruits, other fruits, whole grains/legumes, sweet potato, other potatoes, soybean, nuts, poultry, fish and shrimp, milk, and eggs) and 7 dietary components should be limited (red meat, fried food, refined grains, sugar sweetered beverages, salt, cooking oil, and alcohol). The CPDQS sums up scores from all 22 food groups, with a score range of 0 to 100 points. A higher score indicating better diet quality. Based on Chinese Dietary Guidelines and Chinese Food Pagoda, the present study classified food groups as (1) cereals and tubers; (2) vegetables and fruits; (3) dairy/soy/nuts; (4) animal source food; and (5) condiments/alcoholic beverages [45]. Further details about the scoring criteria for CPDQS are presented in the subsequent paragraph.

One serving is defined as the highest value of the daily recommended intake based on the recommended amounts of various food groups in the Chinese Food Pagoda, with an energy intake of 2000 kcal. In the scoring system for recommended dietary components, the highest score is attained when reaching 60% of the daily recommended intake. The baseline score ranges are as follows: 0 points for 0 servings, 1 point for 0–0.2 servings, 2 points for 0.2–0.4 servings, 3 points for 0.4–0.6 servings, and 4 points for ≥ 0.6 servings. Among these food groups, dark green leafy vegetables, whole grains/legumes, soybean, and fish/shrimp have scoring values that are twice the baseline score, while sweet potatoes and other potatoes have scoring values that are 0.5 times the baseline score. In the scoring system for dietary components should be limited, the highest score is attained when the intake stays below the daily recommended amount. The baseline score ranges are as follows: 4 points for ≤ 1 serving, 3 point for 1–2 servings, 2 points for 2–3 servings, 1 point for 3–4 servings, 0 points for ≥ 4 servings.

For accurate measurement of the CPDQS, this study referenced the common food types and portion sizes provided by the CDG and the Chinese Food Composition Tables [45, 49], along with the price list and actual portion sizes of meals in the refectories of surveyed universities, to annotate the common foods, food portions, and classification criteria for each food group.

SWB was measured by the Index of Well-being (IWB) [50]. The Index of Well-Being (IWB) is a widely used measure to assess SWB of Chinese individuals [51]. It consists of two subsections: the index of general affect and the overall life satisfaction item (α = 0.932). The index of general affect comprises eight items that inquire about the frequency of different emotions experienced, rated on a 7-point scale ranging from 1 (very dissatisfied) to 7 (very satisfied). The overall life satisfaction item is a single question that asks “How satisfied are you with your life as a whole?” and is scored on a 7-point Likert scale (1 = very dissatisfied, 7 = very satisfied). Index of Well-Being = 1.1 * (overall life satisfaction item) + 1.0 * (Index of General Affect).

Demographic covariates included age, gender, registered residence and university information. Health covariates included body mass index (BMI) measured through questions about height and weight. We followed the criteria of the World Health Organization for BMI categories [52]: underweight (BMI ≤ 18.49), normal weight (BMI = 18.50 to 24.90), overweight (BMI = 25.00 to 29.90) and obesity (BMI ≥ 30.00).

### Statistical analysis

Analyses were conducted using IBM SPSS 24 Statistics for Windows. First, frequency analysis and descriptive statistics (n and % for categorical variables, and mean and standard deviation (M ± SD) values for continuous variables) were calculated. Next, to explore the relationships between variables, a correlation analysis over all variables was conducted. The following steps of the multiple regression analyses were conducted to examine the predictive role of CDG adherence and its components on SWB. Specifically, three multiple regression models were built for SWB variable considered as a criterion. Model 1 included only four covariate variables: gender, age, registered residence, and BMI; model 2 added CDG adherence as a simultaneous predictor; and model 3 replaced CDG adherence with its individual components, including cereals/tubers, vegetables/fruits, dairy/soy/nuts, animal source food and condiments/alcoholic beverages. For all analyses a p value of < 0.05 was considered significant.

### Study 2—Daily diary investigation

#### Participants and procedure

Study 2 conducted a daily diary study to confirm the findings of study 1 and enhance the robustness of the test of the proposed relationships because the cross-sectional survey data limited the possibility of causality inferences. In November 2022, using the method of snowball sampling via social-networking, 120 students were recruited from 2 universities located in southern China. The survey was administered online, and participants received the questionnaires through Internet links on a specific social media platform. Participants who wanted to take part in the study were invited to sign a written informed consent form and complete a questionnaire aimed at collecting demographic and baseline data. After completing an initial survey, participants took part in a 10-day daily diary study during which they were requested to complete one daily questionnaire on CDG adherence and daily SWB. The diaries were distributed to participants via Internet links every evening at 8 PM. Subsequently, participants were instructed to complete the daily diary by midnight each day.

Five participants who had insufficient data to calculate time-lagged variables for study analyses were excluded. The final sample for analysis comprised 115 students aged 17 to 24 years (mean age: 19.17 ± 1.13), consisting of 56.5% females. This sample consisted of participants who provided baseline data and a minimum of two daily diaries. In total, the 115 participants provided data for 1020 days out of a potential 1150 days (115 participants×10 days), resulting in a response rate of 88.70%.

### Study variables and measures

The baseline questionnaire was aimed at measuring the demographic covariates and baseline SWB. Demographic covariates included age, gender, registered residence and university information. We measured baseline SWB with the Index of Well-being (IWB) identical to those for Study 1 [50]. Similar to Study 1, we utilized the CPQDS to assess participants’ adherence to the CDG. Furthermore, participants indicated their daily SWB using a single-item measure included in a comprehensive daily emotion assessment adapted from the Affect Valuation Index (AVI) [53]. Participants rated how much they had felt “Happy” over the course of the day on a 7-point scale (1 = not at all; 7 = very much). Previous research commonly employs single-item indicators to measure happiness, demonstrating that measuring SWB with a single item is reliable, valid, and viable [54, 55].

### Statistical analysis

Analyses were conducted using IBM SPSS 24 and Mplus 8.4. First, descriptive statistics and correlation analyses over all variables were calculated by using SPSS 24. In addition, hierarchical multilevel regression was used to examine the hypothesized relationships between CDG adherence and its components and daily SWB. Hierarchical regression allows for within-person and between-person analyses to be conducted simultaneously while adjusting for the autocorrelation of the observations, thus reducing the possibility of inflated results [56]. Hierarchical regression equations were specified using Mplus 8.4, employing restricted maximum likelihood computations. Within-person variables were person-centered, and between-person variables were grand mean-centered.

## Results

### Study 1—the cross-sectional survey

Table 1 shows the general profile of the sample on different aspects of demographics, BMI, CDG adherence and its components, and SWB.

**Table 1:** Descriptive Statistics for Study Variables (Study 1)

Variable	Frequence	%
Gender		
Female	817	57.01
Male	616	42.99
Registered residence		
Rural	677	47.24
Urban	756	52.76
BMI(kg/m²)		
underweight	26	22.61
normal weight	81	70.43
overweight	7	6.08
obesity	1	0.86
	Mean	SD
Age(y)	19.19	1.32
Cereals/Tubers	2.06	0.82
Vegetables/Fruits	1.96	0.81
Dairy/Soy/Nuts	1.81	0.92
Animal source food	1.95	0.65
Condiments/Alcoholic beverages	1.81	0.92
CPDQS	53.31	13.30
SWB	9.21	2.14

**Fig. 1:**
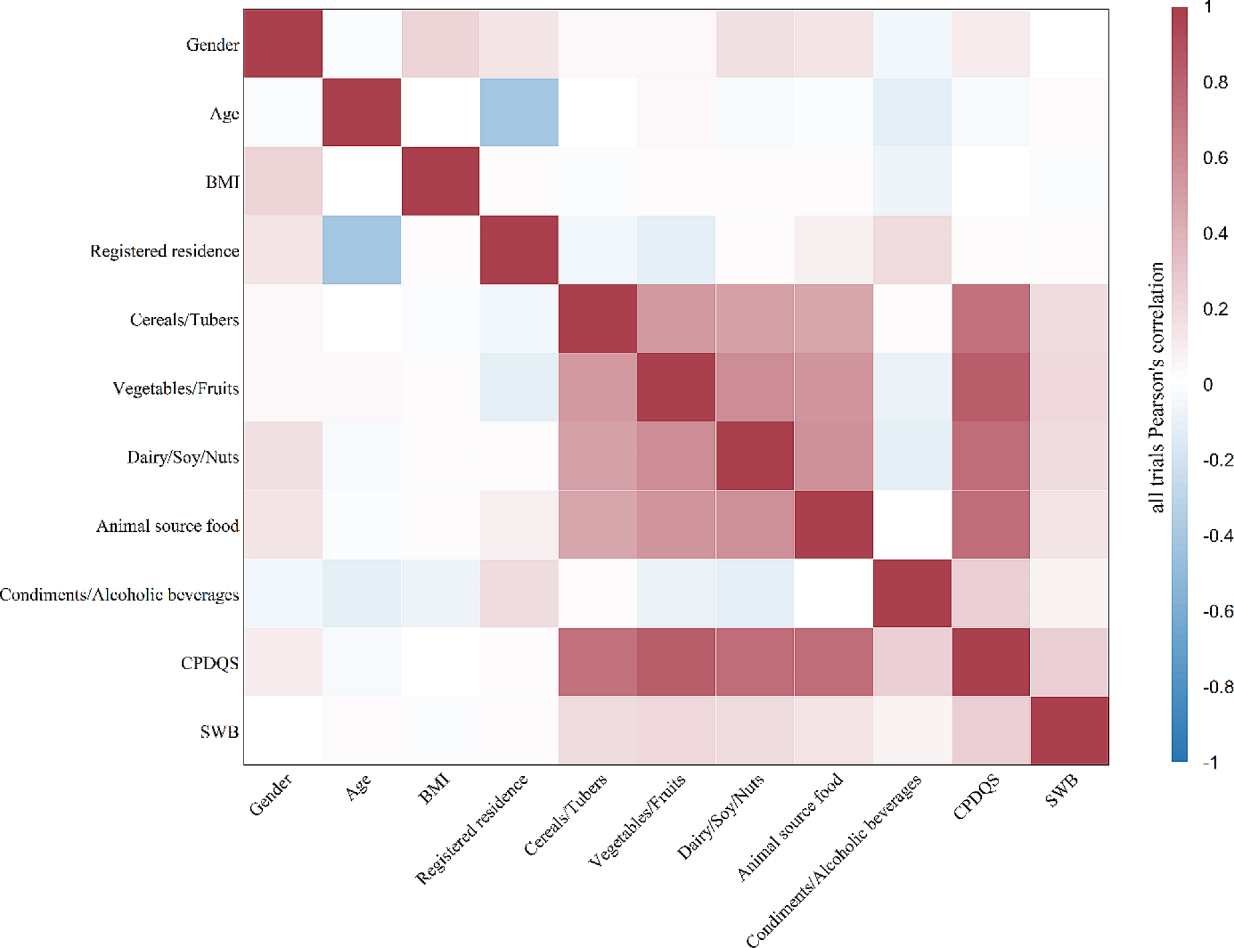
Correlations Between Study Variables (Study 1)

The full pattern of correlations is summarized in Fig. 1. As observed, the primary variables evaluated were significantly positively correlated. Specifically, SWB was positively correlated with CDG adherence (*r* = 0.24, *p*<0.001) and its components including cereals/tubers (*r* = 0.18, *p*<0.001), vegetables/fruits (*r* = 0.12, *p*<0.001), dairy/soy/nuts (*r* = 0.19, *p*<0.001), animal source foods (*r* = 0.15, *p*<0.001), and condiments/alcoholic beverages (*r* = 0.07, *p*<0.01).

**Fig. 2:**
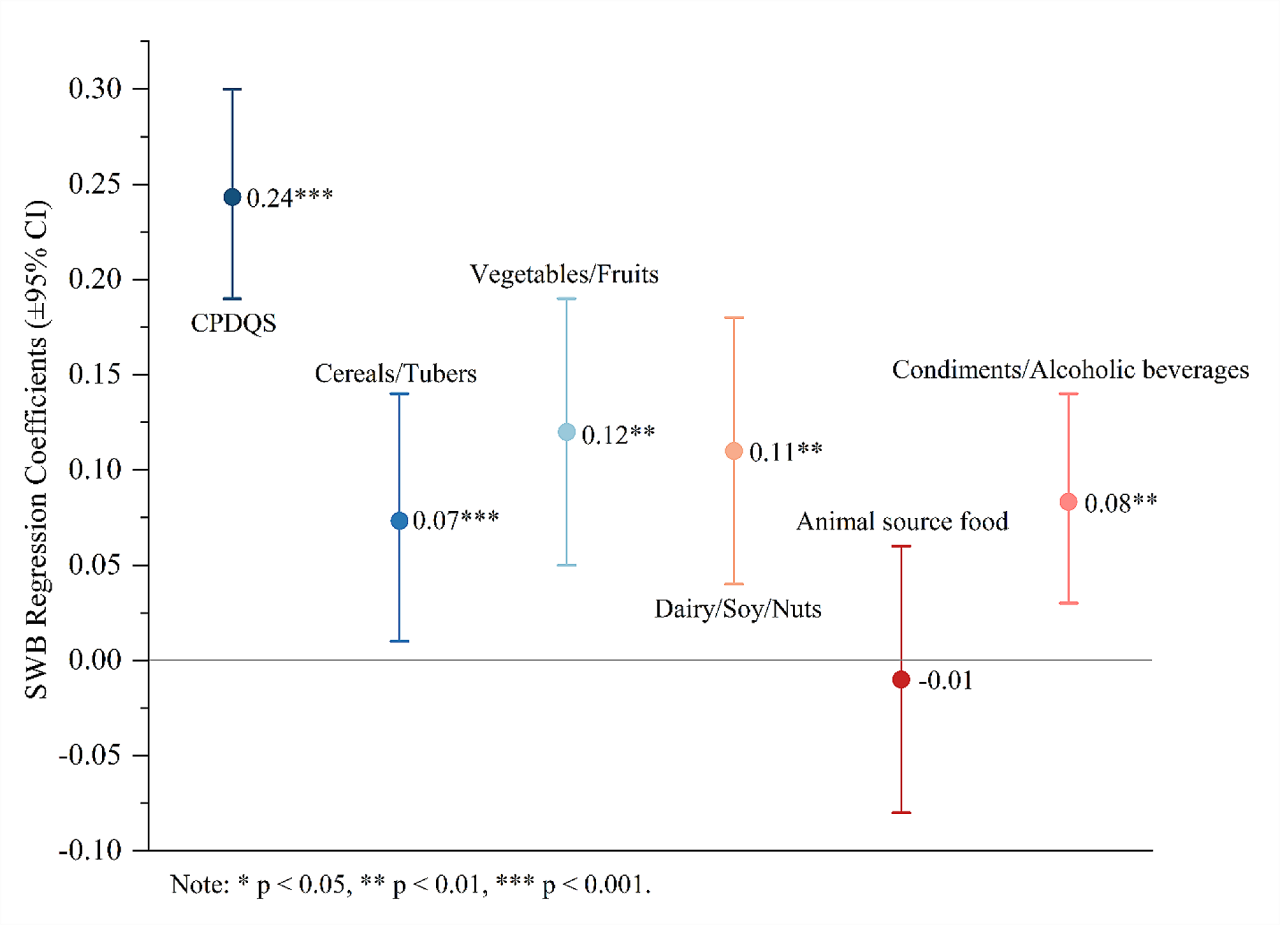
Multivariate regression coefficient plot of CDG adherence and its component effect on SWB (Study 1)

The regression results are presented in Table 2 and visualized in Fig. 2. The results indicated that higher CPDQS (CDG adherence) significantly predicted higher levels of SWB (*β* = 0.24, 95% CI: 0.19–0.30, *p*<0.001). Regarding relationships between SWB and individual components of CDG adherence, four components were unique predictors of happiness: cereals/tubers (*β* = 0.07, 95% CI: 0.01–0.14, *p* = 0.024), vegetables/fruits (*β* = 0.11, 95% CI: 0.05–0.19, *p*<0.001), dairy/soy/nuts (*β* = 0.11, 95% CI: 0.04–0.18, *p* = 0.002), and condiments/alcoholic beverages (*β* = 0.08, 95% CI: 0.03–0.14, *p* = 0.002). Additionally, no significant association was observed between animal source food and SWB (*β*=-0.01, 95% CI: -0.08–0.06, *p* = 0.86).

**Table 2:** Regression Analysis for CPDQS and Its Components Predicting SWB (Study 1)

	Model 1				Model 2				Model 3		
Varible	β	95%CI	t		β	95%CI	t		β	95%CI	t
Gender	0.01	[-0.11, 0.11]	0.07		-0.02	[-0.15, 0.06]	-0.90		-0.02	[-0.15, 0.06]	-0.80
Age(y)	0.04	[-0.02, 0.07]	1.26		0.04	[-0.01, 0.07]	1.44		0.04	[-0.01, 0.08]	1.55
BMI	-0.02	[-0.02, 0.10]	-0.77		-0.02	[-0.02, 0.01]	-0.63		-0.02	[-0.02, 0.01]	-0.62
Registered residence	0.04	[-0.04, 0.19]	1.30		0.04	[-0.04, 0.18]	1.26		0.13	[-0.03, 0.20]	1.43
CPDQS					0.24	[0.19, 0.30]	9.47^***^				
Cereals/Tubers									0.07	[0.01, 0.14]	2.26^***^
Vegetables/Fruits									0.12	[0.05, 0.19]	3.37^**^
Dairy/Soy/Nuts									0.11	[0.04, 0.18]	3.07^**^
Animal source food									-0.01	[-0.08, 0.06]	-0.35
Condiments/Alcoholic beverages									0.08	[0.03, 0.14]	3.15^**^
R²	0.01				0.06^***^				0.06^***^		

Note: * *p* < 0.05, ** *p* < 0.01, *** *p* < 0.001

### Study 2—The daily diary investigation

Table 3 shows the general profile of the sample on different aspects of demographics, BMI, CDG adherence and its components, basic SWB and daily SWB.

**Table 3:** Descriptive Statistics for Study Variables (Study 1)

Variable	Frequence	%
Gender		
Female	65	56.52
Male	50	43.48
Registered residence		
Rural	52	45.22
Urban	63	54.78
BMI(kg/m²)		
underweight	368	25.79
normal weight	942	66.01
overweight	100	7.01
obesity	17	1.19
	Mean	SD
Age(y)	19.17	1.14
Cereals/Tubers	1.91	1.04
Vegetables/Fruits	1.87	0.93
Dairy/Soy/Nuts	1.92	1.10
Animal source food	1.92	0.85
Condiments/Alcoholic beverages	3.35	0.59
CPDQS	54.76	14.14
Basic SWB	9.45	2.24
Daily SWB	9.21	2.14

Table 4 displays descriptive statistics and correlations among all variables of Study 2. Before testing our hypotheses, we examined the focal variables’ within- and between-person variation. For the CDG adherence, cereals/tubers, vegetables/fruits, dairy/soy/nuts, animal source food, condiments/alcoholic beverages, and daily SWB the proportions of within-person variance were 27.56%, 50.71%, 38.87%, 56.52%, 49.05%, 43.25%, and 41.75%, respectively, justifying the application of multilevel modeling. Intraclass correlation (ICC) for SWB is 0.58.

**Table 4:** Correlations Between Study Variables (Study 2)

Variable	1	2	3	4	5	6	7	8	9	10	11	12
1.Cereals/Tubers	—	0.36^***^	0.25^***^	0.24^***^	0.10^**^	0.63^***^	0.17^***^					
2.Vegetables/Fruits	0.60^***^	—	0.34^***^	0.40^***^	0.03	0.80^***^	0.21^***^					
3.Dairy/Soy/Nuts	0.41^***^	0.53^***^	—	0.35^***^	-0.07^*^	0.63^***^	0.25^***^					
4.Animal source food	0.43^***^	0.62^***^	0.53^***^	—	0.01	0.67^***^	0.22^***^					
5.Condiments/Alcoholic beverages	0.17	0.02	-0.18	0.07	—	0.23^***^	0.16^***^					
6.CPDQS	0.76^***^	0.88^***^	0.69^***^	0.79^***^	0.23^*^	—	0.32^***^					
7.Daily SWB	0.23^*^	0.24^*^	0.35^***^	0.27^**^	0.19^*^	0.36^***^	—					
8.Gender	-0.01	0.07	0.18	0.19^*^	-0.10	0.11	0.06	—				
9.Age	-0.01	-0.07	0.01	-0.18	-0.17	-0.11	-0.18	0.04	—			
10.BMI	0.10	0.26^**^	0.23^*^	0.21^*^	0.00	0.25^**^	-0.04	0.25^**^	-0.05	—		
11.Registered residence	-0.16	-0.05	-0.04	0.18	0.14	0.01	0.10	0.08	-0.50^***^	-0.04	—	
12.Basic SWB	0.14	0.16	0.23^*^	0.11	0.01	0.19^*^	0.46^***^	0.04	0.08	-0.06	-0.23^*^	—

Note: Correlations below the diagonal represent person-level correlations (*N* = 115). Correlations above the diagonal are day-level correlations (*N* = 1020). Person-level variables in italics. * *p* < 0.05, ** *p* < 0.01, *** *p* < 0.001

**Fig. 3:**
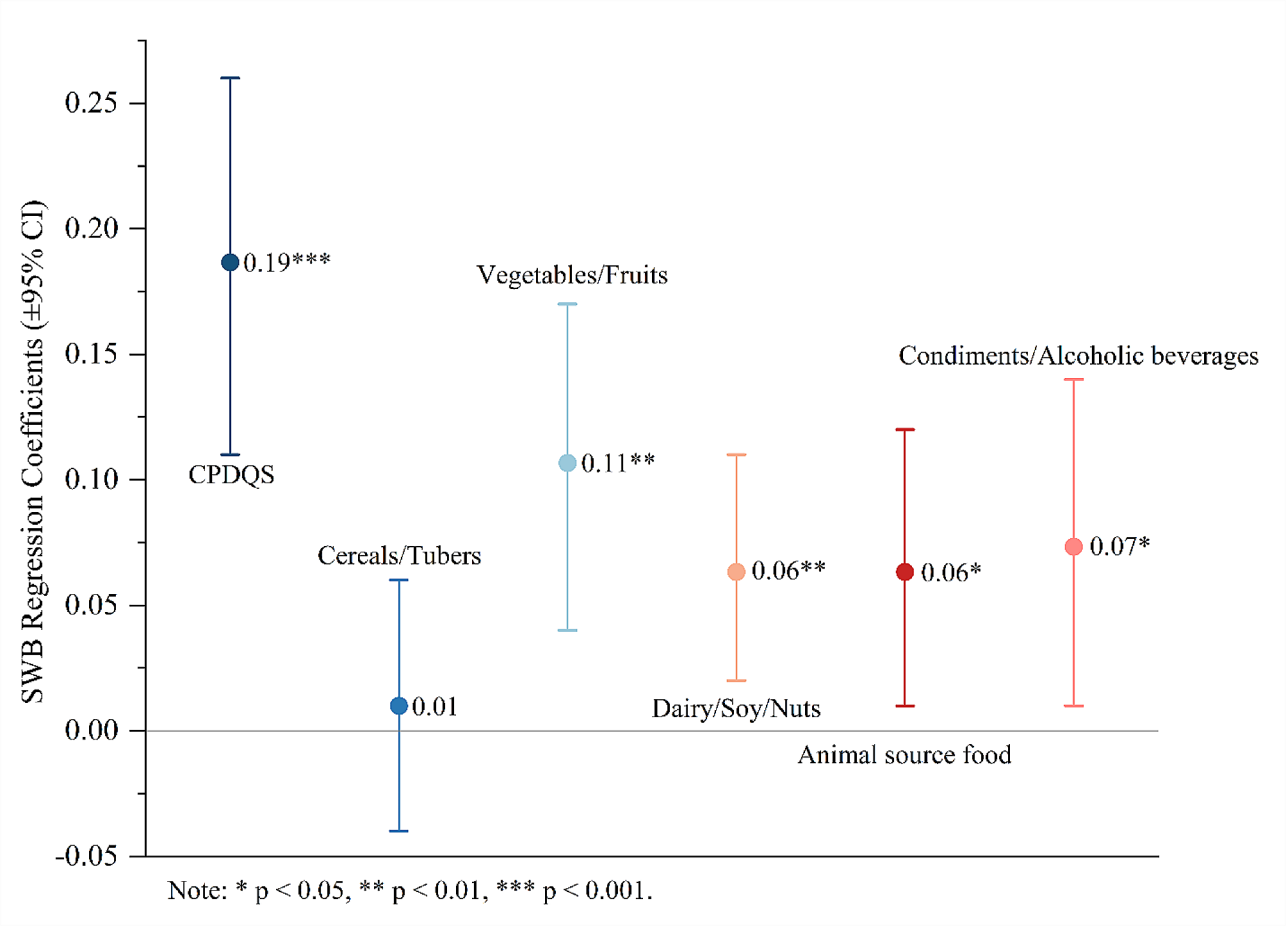
Hierarchical regression coefficient plot of CDG adherence and its component effect on SWB (Study 2)

The hierarchical regression results are presented in Table 5 and visualized in Fig. 3. Model 2 revealed that CDG adherence was positively related to daily SWB (β = 0.19, 95% CI: 0.11–0.26, *p*<0.001) after controlling for demographic covariates and basic SWB. According to model 3, within-subjects effects indicated that when participants reported higher levels of vegetables/fruits (β = 0.11, 95% CI: 0.04–0.17, *p* = 0.001), dairy/soy/nuts (β = 0.06, 95% CI: 0.02–0.11, *p* = 0.009), animal source food (β = 0.06, 95% CI: 0.01–0.12, *p* = 0.026), and condiments/alcoholic beverages (β = 0.07, 95% CI: 0.01–0.14, *p* = 0.026) relative to reported daily SWB. Additionally, no significant association was observed between cereals/tubers and daily SWB (β = 0.01, 95% CI: -0.04–0.06, *p* = 0.670).

**Table 5:** Hierarchical Regression Analysis for CPDQS and Its Components Predicting SWB (Study 2)

	Model 1			Model 2			Model 3		
Varible	β	95%CI	t	β	95%CI	t	β	95%CI	t
Between-person (Level 1)									
Gender	0.06	[-0.16, 0.28]	0.53	0.06	[-0.16, 0.28]	0.53	0.06	[-0.16, 0.28]	0.53
Age(y)	-0.10	[-0.21, -0.01]	-1.79	-0.10	[-0.21, 0.01]	-1.79	-0.10	[-0.21, 0.01]	-1.79
BMI	-0.01	[-0.04, 0.03]	-0.23	0.07	[-0.04, 0.03]	2.08	-0.01	[-0.04, 0.03]	-0.23
Registered residence	0.20	[-0.07, 0.46]	1.46	0.20	[-0.07, 0.46]	1.46	0.20	[-0.07, 0.46]	1.46
Basic SWB	0.16	[0.10, 0.22]	5.57^***^	0.16	[0.02, 0.15]	5.57^***^	0.16	[0.10, 0.22]	5.57^***^
Within_person (Level 2)									
CPDQS				0.19	[0.11, 0.26]	4.76^***^			
Cereals/Tubers							0.01	[-0.04, 0.06]	0.43
Vegetables/Fruits							0.11	[0.04, 0.17]	3.23^**^
Dairy/Soy/Nuts							0.06	[0.02, 0.11]	2.60^**^
Animal source food							0.06	[0.01, 0.12]	2.22^*^
Condiments/Alcoholic beverages							0.07	[0.01, 0.14]	2.23^*^
R²		0.10^***^			0.17^***^			0.22^***^	

Note: * *p* < 0.05, ** *p* < 0.01, *** *p* < 0.001

## Discussion

In light of the shifting focus toward a more positive and well-being-centered approach to eating behavior [57, 58], it becomes crucial to gather evidence on modifiable lifestyle behaviors and interventions that have a favorable impact on SWB. To the best of our knowledge, the present study is the first to evaluate the association between adherence to the Chinese dietary guidelines and SWB among a large representative sample of Chinese adults. Based on the findings of a cross-sectional survey and a daily diary investigation, the results indicate that participants with greatest adherence to the CDG reported higher levels of SWB compared to those with the lowest adherence. Our findings provide novel information and supportive data for an association between high dietary quality and positive psychological well-being.

Regarding the relation between CDG adherence and SWB, previous studies have linked healthy dietary patterns to the SWB [26, 40, 43]. Consistent with our findings, Moreno et al. reported that a high adherence to a healthy dietary pattern, resembling the Mediterranean dietary pattern, was associated with reduced odds of experiencing depressive episodes and improved SWB [43]. The CDG emphasizes the importance of consuming appropriate quantities and proportions of various food types, advocating a well-balanced dietary structure [45]. Similarly, results from a comprehensive systematic review revealed that adopting a healthy and balanced diet has the potential to enhance individuals’ overall well-being and improve quality of life [59]. Although the precise mechanisms remain uncertain, the protective links of CDG adherence with SWB may be attributed to its nutrient composition and psychological impact. On the one hand, the influence of specific nutrients found in the characteristic foods of the CDG on brain function provides a plausible explanation for the obtained results [40, 60]. Specifically, the inclusion of foods such as fruits, vegetables, legumes, and whole grains in the CDG provides essential nutrients known to be associated with a high concentration of brain serotonin [61]. Serotonin, a neurotransmitter closely linked to happiness, positive mood, and motivation, plays a significant role in promoting feelings of vitality and psychological well-being [61, 62]. On the other hand, the relationship between CDG adherence and SWB may be purely perceptual, resulting from consumers’ attribute beliefs and expectations associated with health food. According to the food well-being paradigm, feelings of satisfaction and goal achievement derived from making healthy food choices may contribute to experiencing emotional well-being [28, 63]. Therefore, participants who exhibit high levels of adherence to CDG may infer that their nutrition are healthy, and as a result, the feeling of satisfaction derived from making dietary choices may contribute to the experience of SWB. In line with this, a positive perception of health has been consistently associated with both life satisfaction and happiness, as evidenced by a wide range of empirical studies [64–66].

Moreover, our findings also revealed the consumption of certain components of the CDG is positively associated with SWB. In particular, a cross-sectional survey and daily diary investigation consistently show that higher intake of vegetables/fruits and dairy/soy/nuts is positively associated with increased levels of SWB. Conversely, decreasing the consumption of condiments/alcoholic beverages has been linked to improved SWB. These results are consistent with previous studies showing that consuming a significant quantity of vegetables and fruits is associated with increased levels of self-efficacy and happiness [27, 29, 67], while concurrently lowering levels of psychological distress [68, 69]. In addition, a cross-sectional survey also identified a positive association between higher consumption of dietary protein sources, including dairy products, soy, and nuts, with improved quality of sleep and greater happiness [70]. Regarding the SWB effects of condiments and alcoholic beverages, previous studies have revealed that diets low in salt, sugar, and fat lead are associated with higher levels of SWB [71, 72]. Additionally, excessive alcohol consumption has shown a negative correlation with eudaimonic well-being and is a predictor of psychological distress [58, 73, 74]. To the best of our knowledge, the current evidence concerning these findings is both limited and indirect. Consequently, further research is imperative to provide clarity.

Surprisingly, the cross-sectional survey observed a significant positive association between the consumption of cereals/tubers and SWB, while no such association was identified in daily diary investigation. Consuming cereals and tubers, which are rich in dietary fiber and serve as excellent sources of vitamin B and carbohydrates, is integral to maintaining a healthy lifestyle [45]. The CDG recommends daily intake of whole grains and tubers, while cautioning against excessive consumption of refined grains such as rice and flour [45]. Supporting the results of the cross-sectional survey, cereal crops can serve as a protective factor beneficial for the mental health of adults across previous studies [75, 76]. Further, the distinct nutrients present in cereals and tubers are also linked to a substantial concentration of brain serotonin [40]. However, the ten-day diary survey did not uncover the daily fluctuations in cereal and tuber consumption were associated with SWB. A tentative explanation for the inconsistency in the relationship between the cross-sectional survey and the daily diary investigation has been lies in cultural specificity in diet [77, 78]. The Chinese population has traditionally followed a dietary pattern centered around grains, with refined grains such as rice and wheat serving as the primary staples for all three meals [79]. Cultural differences in dietary habits among Chinese individuals may contribute to a weaker perception of the association between refined grains and health. As a result, the impact of daily fluctuations in cereal/tuber consumption on SWB may be diminished [77, 80].

On the other hand, the daily diary investigation has observed a significant positive association between the consumption of animal source food and SWB, whereas no such association has been identified in the cross-sectional survey. The CDG advocated for increasing the consumption of aquatic products, such as fish and shrimp, and reducing the excessive intake of red and processed meat products [45]. Previous research on the association between animal food sources and SWB have been inconsistent [43, 81, 82]. Some studies have found a relationship between reduced intake of red and processed meat or increased fish consumption and well-being [40, 82, 83], while other studies have not found such a correlation [1, 43]. Although the relationship appears to be more complex than anticipated, a tentative explanation is the conflict between the rational and emotional selves [84, 85]. Within the realm of diet, the emotional self exhibits a positive response to red and processed meat, leading to instant gratification. The rational self, in contrast, acknowledges the significance of healthy behaviors like a nutritious diet and regular exercise, resulting in delayed gratification [1]. Therefore, restricting red and processed meat consumption may reduce immediate gratification in cross-sectional studies, but it may give us deliberative well-being in a ten-day diary investigation.

The results of this study must be interpreted taking into account some limitations. Firstly, the limited sample size of our study, which include only college students from southern China, restricts the generalizability of our research findings. Hence, it is imperative for future research to replicate the findings using a more heterogeneous and broader sample. Secondly, although two studies were conducted using different samples and methods, the characteristics of a survey restrict the possibility of causal inferences. To extend this research and verify the directionality of these findings, incorporating an experimental design or randomized controlled trial could help determine whether CDG adherence is associated with increased happiness over time. Additionally, the study utilizes subjective reports to evaluate SWB, and the outdated nature of the instruments results in certain limitations. Future research would benefit from employing multiple measurements of SWB using a variety of indicators and integrating physiological health measures. This approach would address the significant dearth of research on the impact of healthy eating on SWB and yield evidence-based findings to determine whether the influence of healthy eating is a placebo effect or an additional mediator with physical health benefits. Finally, the study omits an exploration of factors beyond a healthy diet that could impact SWB. Existing research suggests that social and lifestyle factors, including the quality of social relationships, income, smoking, sleep, and physical activity, also play a role in predicting SWB [86, 87]. Future studies on the connection between a healthy diet and SWB should include controls for these variables to confirm the association.

## Conclusions

This research analyzed the influence of food consumption on SWB with two studies: a cross-sectional survey and a daily diary investigation. Greater adherence to the CDG was associated with better SWB in a representative sample of Chinese adults. Additionally, the consumption of certain food groups of CDG such as vegetables/fruits, dairy/soy/nuts, and condiments/alcoholic beverages is also correlate with SWB. In conclusion, the assessment and recommendation to adhere to the CDG could serve as an ecological approach for enhancing the affective experience and life evaluation of the population. This approach not only promotes SWB but also offers additional benefits that extend beyond the realm of well-being.

## Data Availability

The datasets used and/or analyzed during the current study are available from the corresponding author on reasonable request.
